# Cemento-Osseous Dysplasia of the Jaw: Demographic and Clinical Analysis of 191 New Cases

**DOI:** 10.3390/dj11050138

**Published:** 2023-05-19

**Authors:** Katherine Decolibus, Shokoufeh Shahrabi-Farahani, Anmol Brar, Shane D. Rasner, Sarah E. Aguirre, Adepitan A. Owosho

**Affiliations:** 1 Department of Diagnostic Sciences, College of Dentistry, The University of Tennessee Health Sciences Center, Memphis, TN 38163, USA; 2 Department of Otolaryngology—Head & Neck Surgery, College of Medicine, The University of Tennessee Health Sciences Center, Memphis, TN 38163, USA

**Keywords:** florid cemento-osseous dysplasia, periapical cemento-osseous dysplasia, focal cemento-osseous dysplasia, benign fibro-osseous lesion

## Abstract

Cemento-osseous dysplasia (COD) is a form of benign fibro-osseous lesion of the jaw. We sought to evaluate the demographic and clinical presentations of COD by collecting and analyzing the demographic, clinical, radiographic, and pathologic data of COD diagnosed in our institution from 2017 to 2022. Over this six-year period, the records of 191 patients with COD were reviewed. Most patients were African American and female. Eighty-five patients were diagnosed with florid COD (FLCOD), 63 with periapical COD (PCOD), and 43 with focal COD (FCOD). Twenty-eight (14.7%) patients presented symptoms. The most common symptom was pain. All the symptomatic cases of COD that were histopathologically diagnosed were osteomyelitis in the setting of COD. Symptomatic patients were older (mean of 61.3 years) than the asymptomatic patients (mean of 51.2 years). Due to the radiographic appearance of a radiolucency or a mixture of radiolucency and radiopacity, forty-five asymptomatic patients were biopsied. Most of the asymptomatic patients biopsied were patients with FCOD (*n* = 19, 54.3%), followed by PCOD (*n* = 16, 25.8%), and FLCOD (*n* = 10, 15.2%). FLCOD is the most common form of COD to present with symptoms. Due to the significant overlap in clinical and radiographic presentation with other entities, FCOD and PCOD remain a diagnostic challenge to dentists. In conclusion, we analyzed the demographic and clinical features of 191 new cases of COD, which reaffirms that cemento-osseous dysplasia is a condition that primarily affects middle-aged females of African descent and occurs more frequently in the mandible.

## 1. Introduction

Cemento-osseous dysplasia (COD) is classified as a benign fibro-osseous lesion confined to the tooth-bearing/periapical regions of the jaw [1,2,3]. COD is characterized by the replacement of normal bone with fibrous and cementoid tissue. In the past, COD was considered a non-neoplastic, idiopathic, reactive, or hamartomatous process arising from the apical periodontium due to its close association with the apices of teeth and histologic resemblance to cementum. Recently, pathogenic hotspot mutations were detected in the RAS-MAPK signaling pathway, with mutations of *BRAF*, *HRAS*, *KRAS*, *NRAS*, and *FGFR3* in COD [4].

There are three types of COD differentiated by the location and extent of the lesion: periapical COD (PCOD) is limited to the apical region of a few adjacent teeth in the anterior mandible, focal COD (FCOD) is limited to the apical region of a single tooth of the posterior jaw, and florid COD (FLCOD) is more extensive, with multifocal and multi-quadrant involvement of the jaws. COD occurs more commonly in the mandible, with a predilection for middle-aged females of African descent [2,5,6]. Clinically, COD is usually asymptomatic, with both PCOD and FCOD often identified as an incidental radiographic finding [2,5,6]. FLCOD may present with symptoms such as pain and exudate when secondarily infected, resulting in osteomyelitis [5,6,7,8]. Radiographically, in the early stage of maturation, COD may present as a radiolucent lesion, then a mixed radiopacity, and later as a radiopaque lesion with a radiolucent rim [9].

Clinical and radiographic features may be sufficient to arrive at a diagnosis of COD without pathologic confirmation [2,10]. However, an early radiolucent FCOD/PCOD may be confused with a periapical granuloma/cyst [11,12,13]. Distinguishing FCOD/PCOD from cemento-ossifying fibroma may be challenging [2,3]. Other radiographic differential diagnoses such as idiopathic osteosclerosis, condensing osteitis, exostoses, hypercementosis, cementoblastoma, and medication-related osteonecrosis of the jaw (MRONJ) are discussed. This study aims to analyze the demographic and clinical features of a relatively large series of CODs.

## 2. Materials and Methods

A six-year retrospective analysis was performed by retrieving the clinical, radiographic, and pathologic records of patients diagnosed with COD from the electronic health record of the University of Tennessee Health Science Center (UTHSC), College of Dentistry, from 1 January 2017 to 31 December 2022. The study was approved by the UTHSC IRB #(23-09328-XM).

To confirm the diagnoses, radiographs (periapical (full mouth series) and panoramic) of all patients with the term “cemento-osseous dysplasia” or “osseous dysplasia” were evaluated using the WHO classification criteria. The radiographs were made for baseline, diagnosis, and treatment planning as the patients initially entered our dental school clinical program. All radiographs were reviewed by the senior author (AAO), a board-certified oral and maxillofacial pathologist. Pathology reports of all cases that were biopsied were reviewed. The following clinical information was retrieved and analyzed descriptively: age at diagnosis, gender, ethnicity, location, and presenting symptoms. The radiographic features of PCOD and FCOD were categorized as either completely radiolucent, mixed-radiopacity, or radiopaque ± a radiolucent rim.

## 3. Results

### 3.1. Demographic Characteristics

Between 1 January 2017 and 31 December 2022, there were 442 patient records with the terms “cemento-osseous dysplasia” or “osseous dysplasia” noted. In that group, after the radiographs were evaluated, 191 patients were confirmed to have diagnosis of COD (128 radiographic alone, 50 histopathologic alone, and 13 both radiographic and histopathologic). The ages ranged from 18 to 94 years, with a mean of 52.7 years. COD was most prevalent in the 6th decade of life. There were 160 (83.8%) African American (AA), 24 (12.6%) Caucasian (C), 4 (2.1%) Hispanic (H), and 3 (1.6%) Asian (A) patients.

There were 178 (93.2%) female and 13 (6.8%) male patients. The average age of the male patients was 51.2 years (range: 18–86 years). The ethnicities of the male patients were: nine (69.2%) AA, two (15.4%) C, and two (15.4%) A. The average age of the female patients was 52.7 years (range: 18–94 years). The ethnicities of the female patients were: 151 (84.8%) AA, 22 (12.4%) C, 4 (2.2%) H, and 1 (0.6%) A.

#### Types of COD

**Figure 1:**
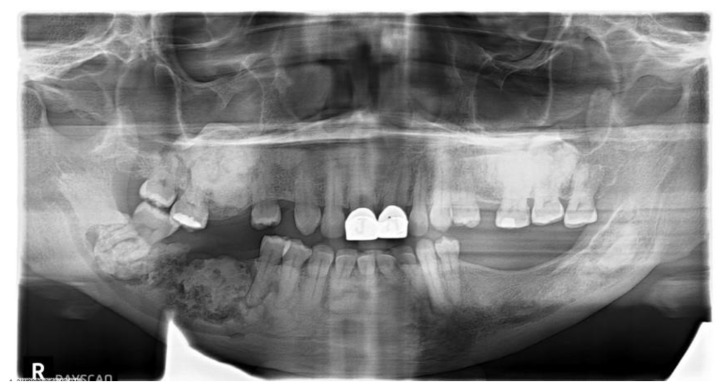
Florid cemento-osseous dysplasia involving all four quadrants of the jaws in a symptomatic 54-year-old African American female.

**Figure 2:**
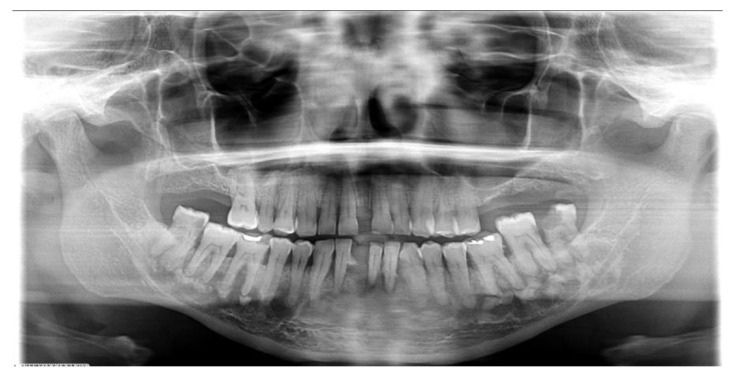
Florid cemento-osseous dysplasia involving both quadrants of the mandible in an asymptomatic 48-year-old African American female.

**Figure 3:**
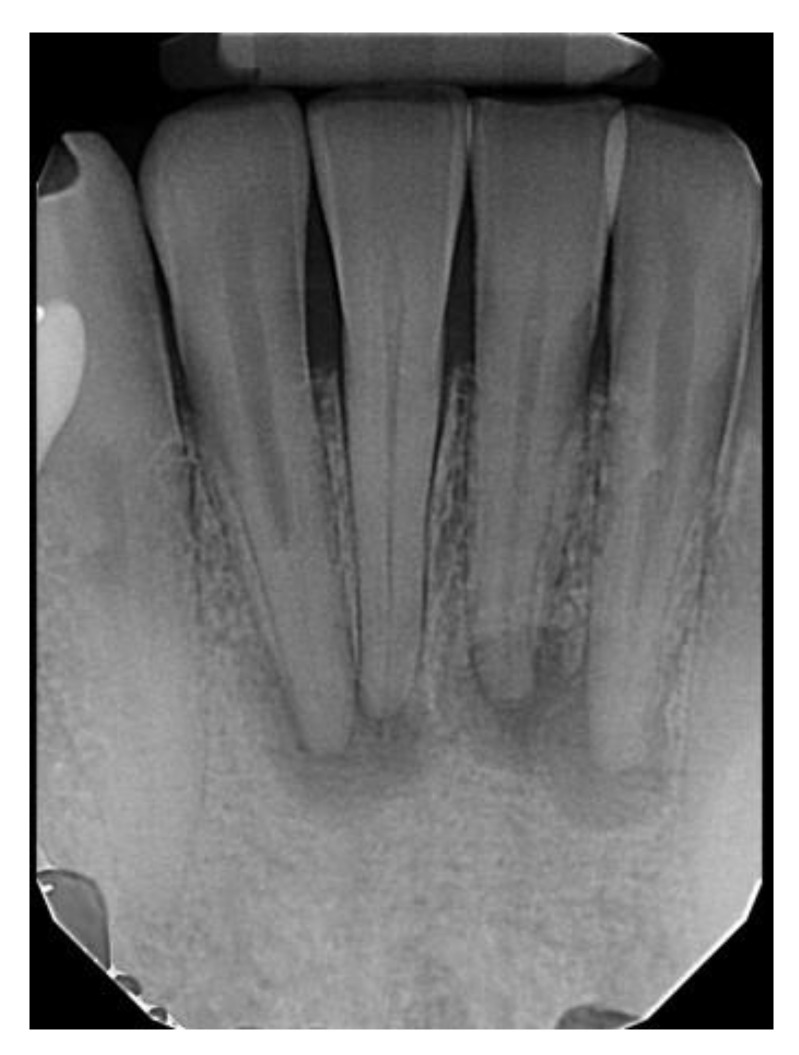
Periapical cemento-osseous dysplasia involving the incisors of the mandible in an asymptomatic 52-year-old Caucasian male.

**Figure 4:**
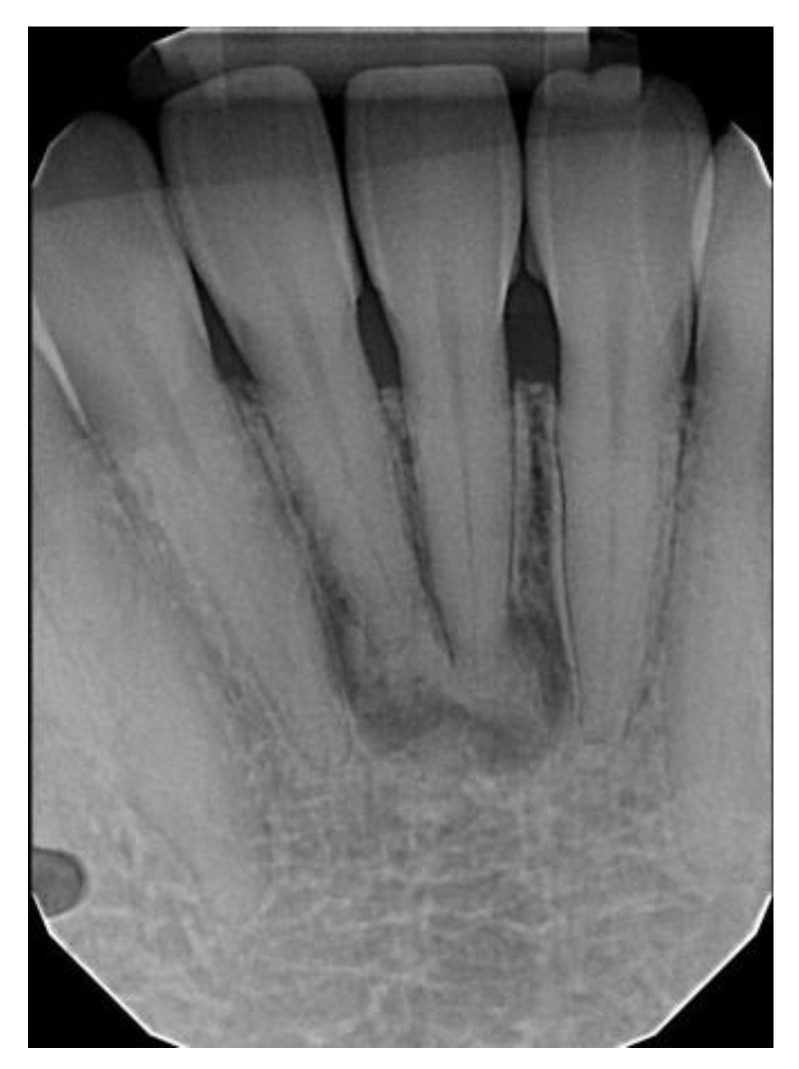
Periapical cemento-osseous dysplasia involving the central incisors of the mandible in an asymptomatic 45-year-old African American female.

**Figure 5:**
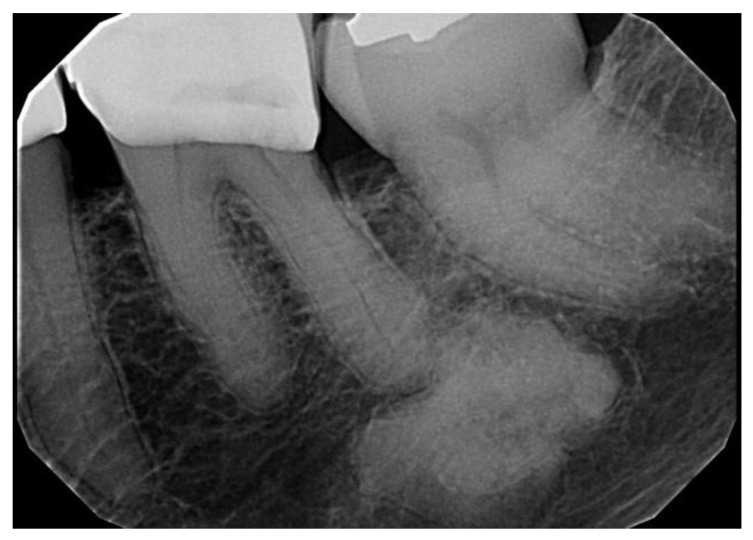
Focal cemento-osseous dysplasia involving a molar of the mandible in an asymptomatic 69-year-old African American female.

**Figure 6:**
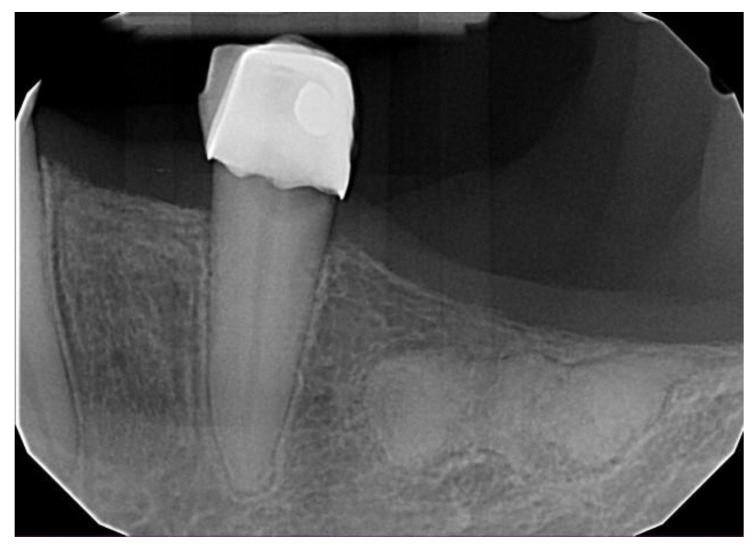
Focal cemento-osseous dysplasia involving the posterior edentulous area of the mandible in an asymptomatic 63-year-old African American female.

In our study, FLCOD comprised most of the COD cases, with 85 (44.5%) patients (Figure 1 and Figure 2) (63 cases had radiographs for review). This was followed by PCOD, with 63 (33%) patients (Figure 3 and Figure 4), and FCOD, with 43 (22.5%) patients (Figure 5 and Figure 6). There were 82 (96.5%) female patients and 3 (3.5%) male patients with FLCOD. The average age of the patients with FLCOD was 58.5 years (range: 26–94 years). FLCOD was the most prevalent in the 7th decade of life. The cases of FLCOD were all seen in AA patients. The mandible was involved in 82 of the 85 patients, with concomitant involvement of the maxilla in 23 patients, and involvement of the maxilla alone in 3 patients.

There were 55 (87.3%) female and 8 (12.7%) male patients with PCOD. The average age of these patients was 48.3 years (range: 25–83 years). PCOD was the most prevalent in the 4th decade of life. The ethnicities of these patients were: 53 (84.1%) AA, 7 (11.1%) C, 2 (3.2%) A, and 1 (1.6%) H. The periapical region of the anterior mandible was involved in all PCOD cases. Of the 52 cases with radiographs to review, 14 (27%) were completely radiolucent, 25 (48%) were mixed-radiopacity, and 13 (25%) were radiopaque ± a radiolucent rim.

There were 41 (95.3%) female and 2 (4.7%) male patients with FCOD. The average age of the patients with FCOD was 47.7 years (range: 18–88 years). FCOD was the most prevalent in the 6th decade of life. The ethnicities of these patients were: 22 (51.2%) AA, 17 (39.5%) C, 3 (7%) H, and 1 (2.3%) A. The periapical region of the posterior mandible was involved in all FCOD cases. Of the 24 cases with radiographs to review, 2 (8.3%) were completely radiolucent, 9 (37.5%) were mixed-radiopacity, and 13 (54.2%) were radiopaque ± a radiolucent rim. A summary of the demographic features of patients with cemento-osseous dysplasia is presented in Table 1.

### 3.2. Clinical Features

Twenty-eight (14.7%) patients presented with the following symptoms: isolated bone pain, pain and swelling, discharge, exposed bone, non-healing extraction site, and a failed implant. A summary of the presenting symptoms is presented in Table 2. To rule out dental infection as the etiology, a pulp vitality test was performed on all associated teeth, as routinely performed in the school’s clinic. The patients were all female, 23 AA and 4 C. Nineteen symptomatic patients were diagnosed with FLCOD, eight with FCOD, and one with PCOD. The age of the patients with symptoms ranged from 41 to 84 years, with a mean of 61.3 years. The mandible was the location in 26 patients and the maxilla in 2 patients, with symptoms of draining fistulae and non-healing extraction sites. One case of symptomatic FCOD was associated with an impacted mandibular third molar and an inflamed dentigerous cyst.

**Figure 7:**
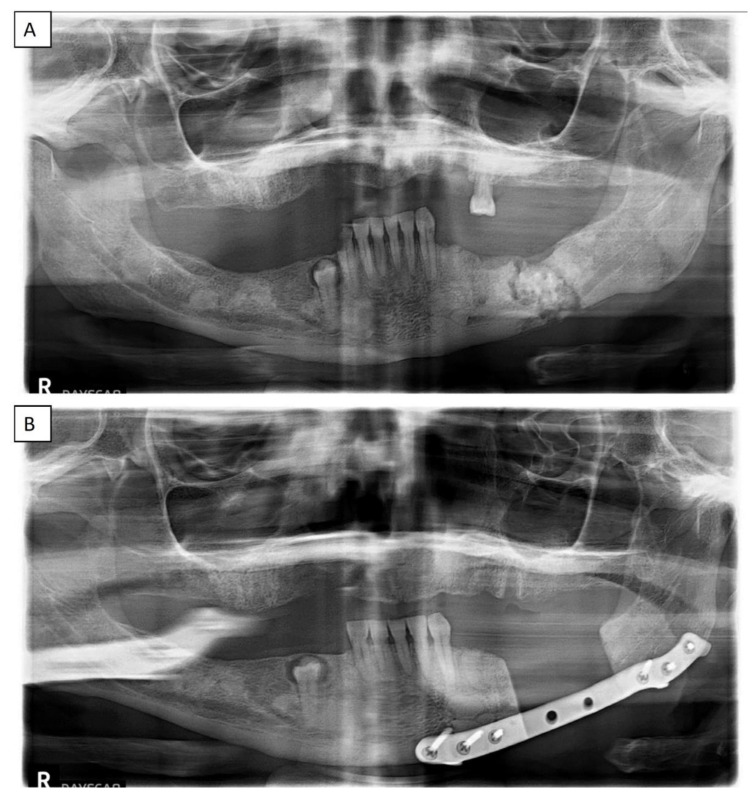
Florid cemento-osseous dysplasia involving both quadrants of the mandible in a symptomatic 63-year-old African American female (**A**). Post-resection and reconstruction radiograph (**B**).

**Figure 8:**
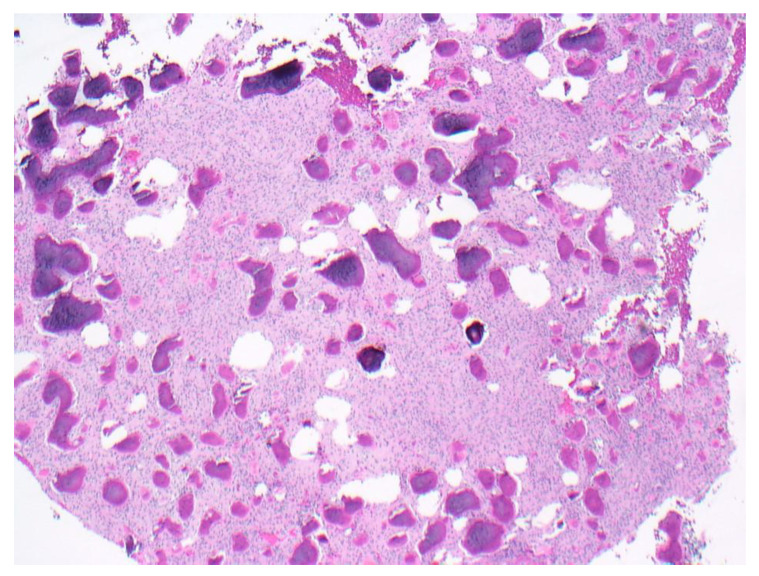
Photomicrograph of cemento-osseous dysplasia showing a mixture of irregular bony trabecular, woven bone, and cementum-like structures in a spindled-to-plump connective tissue stroma.

The lesions of all patients with symptoms that were biopsied were diagnosed as osteomyelitis in the setting of a COD. One of those cases was managed with mandibular surgical resection (Figure 7A,B). Due to the radiographic appearance of radiolucency or a mixture of radiolucency and radiopacity, 45 asymptomatic patients were also biopsied. The majority of the asymptomatic patients biopsied presented with FCOD (*n* = 19, 54.3%), followed by PCOD (*n* = 16, 25.8%), and FLCOD (*n* = 10, 15.2%). Histopathologically, CODs presented as a mixture of irregular bony trabecular, woven bone, and cementum-like structures in a spindled-to-plump connective tissue stroma with no capsule (Figure 8).

## 4. Discussion

COD is classified as a benign fibro-osseous lesion (BFOL) confined to the tooth-bearing/periapical regions of the jaw. BFOLs of the craniofacial bones encompass a diverse range of lesions, sharing similar histopathologic features, but with distinctive clinical and imaging characteristics. They are characterized by the replacement of normal bone by fibrous tissue containing osteoid and cementoid tissue. These lesions include fibrous dysplasia, ossifying fibroma, juvenile ossifying fibroma (psammomatoid and trabecular), gigantiform cementoma, and COD (florid, periapical, and focal). Although these lesions are classified as BFOLs, they do not share the same etiopathogenesis and they appear to be characterized by a heterogenous molecular profile. Fibrous dysplasia is etiologically associated with mutation in *GNAS* [14], whereas the dysregulation of the Wnt and NOTCH pathways has been implicated in the etiopathogenesis of ossifying fibroma [15,16]. *MDM2* amplification has been reported in craniofacial BFOLs, especifically in juvenile ossifying fibromas and ossifying fibromas [17]. Inactivating mutations in *CDC73*, a tumor suppressor gene, have been reported in cases of ossifying fibroma in the setting of hyperparathyroidism-jaw tumor syndrome and occasionally in sporadic cases of ossifying fibroma [18]. Next-generation sequencing of craniofacial ossifying fibroma revealed alterations in *FOS*, *FOSB*, *COL1A1*, and *TBX3* genes [19]. *SATB2* translocations and *SETD2* mutation have been reported in psammomatoid juvenile ossifying fibroma [20,21]. Mutations in *ANO5* have been described in gigantiform cementoma [22], and recently, mutations of *BRAF*, *HRAS*, *KRAS*, *NRAS*, and *FGFR3* were described in COD [4].

Using our institution’s electronic health records from 2017 to 2022, we conducted a clinical analysis of 191 cases with a confirmed diagnosis of COD of the jaw. Our study documents COD as a pathologic condition with a predilection for females (93.2%) and middle-aged (6th decade) individuals of African descent (83.8%). However, among the patients with FCOD, the ethnic background was relatively balanced between 51.2% AA and 39.5% C. These demographic features are in accordance with previously reported literature [2,3,6]. The studies by Summerlin and Tomich, Su et al., Alsufyani and Lam, Owosho et al., and Kawai et al. all reported a female predilection (82.9–94.3%) [2,3,5,6,7]. Studies that analyzed ethnicity also reported a predilection for individuals of African descent [2,6]. The most common type of COD in this study was FLCOD (44.5%). However, as reported in similar studies, if both PCOD and FCOD are combined, they make up the most common type of COD [2,3,5,6].

As also noted in similar studies, most patients in this study (85.3%) were asymptomatic. The study by Summerlin and Tomich reported that all FCODs were asymptomatic lesions, usually identified on radiographic examination [3]. The study by Su et al. reported that 63% of their FCOD patients were asymptomatic and FCOD was identified during routine radiographic examination [2]. The study by Alsufyani and Lam reported that 72.2% of their COD patients were asymptomatic, and the study by Owosho et al. reported that 77.1% of their COD patients were asymptomatic [5,6]. However, the study by Kawai et al. reported that 59% of their patients were symptomatic [7]. It has been suggested that older patients with COD are more likely to present with one or more symptoms compared to younger patients, who are typically asymptomatic [5,23]. In this study, symptomatic patients were older (mean of 61.3 years) than the asymptomatic patients (mean of 51.2 years). Jaw pain was the most common presenting symptom in every symptomatic case histopathologically diagnosed as osteomyelitis in the setting of COD. Osteomyelitis in the setting of COD has been attributed to the maturation of the lesion, associated with a progressive production of avascular cementum-like calcification. This increases the susceptibility of the lesion to infections. Due to its avascular nature, the lesion may not respond well to antibiotic therapy, thus necessitating the removal of the sequestrum [24,25]. In this current study, two of the symptomatic patients presented with post-extraction complications of non-healing sockets, and pain and swelling in the setting of FLCOD. Waldron et al. recommended that tooth extraction should be avoided in patients with FLCOD, as many patients presented with poor socket healing and sequestrum formation following extraction [26]. Additionally, one of the symptomatic patients presented with a failed implant placement in the setting of FLCOD. It has been suggested that placing an implant in a jaw with FLCOD poses a risk of implant failure and osteomyelitis because the pathophysiology of COD and the implant may serve as a communication pathway for oral flora to access the avascular cementum-like calcification [6]. COD has been reported in the literature to be associated with simple bone cysts [5,27,28]. In this current study, one symptomatic case of FCOD was associated with an impacted mandibular wisdom tooth and an inflamed dentigerous cyst.

It is of interest that many of the patients (45/165 (27.3%)) with FCOD and PCOD, though asymptomatic, were biopsied. This is most likely due to the diagnostic conundrum that PCOD and FCOD pose [2,3,6]. A radiolucent lesion of either FCOD or PCOD may be confused with an odontogenic periapical lesion such as a periapical granuloma/cyst, which would typically be non-vital on pulp testing of the associated tooth [11,12,13]. However, if FCOD or PCOD are present, a vital pulp test may prompt the clinician to investigate further to reach a definitive diagnosis. In this study, 27% of PCOD and 8.3% of FCOD presented with completely radiolucent lesions.

In addition, distinguishing FCOD from ossifying fibroma (OF) may be challenging. A combination of clinical, radiographic, histopathologic, and even intraoperative findings may be required to arrive at a confident definitive diagnosis [2]. Both entities have a predilection for the posterior mandible; however, the majority of OFs are not associated with the apices of teeth [2]. When occurring in radicular areas, OF frequently demonstrates root divergence or the displacement of the involved teeth, a feature not observed in FCOD [2]. Both entities may present as well-circumscribed, with or without a sclerotic border, radiolucent, or mixed radiolucent–radiopaque radiographic lesions [2,29]. A primarily radiopaque lesion with a thin radiolucent rim may be seen in late-stage FCOD, but is rarely observed in OF [2]. In the absence of secondary simple bone cyst formation, clinical expansion is uncommon in all forms of COD, while the neoplastic nature of OF may result in significant clinical expansion [2,29]. Intraoperative findings may provide valuable diagnostic information, as OF is often removed in single or large enucleated fragments, whereas COD is removed in smaller, numerous, hemorrhagic fragments [2,29]. There is a significant histopathologic overlap between the two entities, but Su et al. reported some features as statistically significant between them. Thick, curvilinear, “ginger root” trabeculae within the center of the specimen are more frequently observed in FCOD [2]. A cellular, storiform stromal pattern is more commonly observed in OF [2]. Additionally, free hemorrhage throughout the lesion with sinusoidal vascular spaces approximating the bony trabeculae are features associated with FCOD [2]. FCOD does not require further treatment once a diagnosis is established, while OF may require further surgical intervention for the complete removal [29,30].

Other radiographic differential diagnoses to consider are idiopathic osteosclerosis, condensing osteitis, exostoses, hypercementosis, cementoblastoma, and MRONJ. Idiopathic osteosclerosis is also known as a dense bone island or enostosis [31]. It is an asymptomatic focal radiopacity that is not associated with any inflammatory, dysplastic, neoplastic, or systemic condition [31]. It is an incidental finding on radiographs and can be located anywhere in the jaw. Radiographically, it appears as a well-defined homogenous radiopacity with no radiolucent rim. Condensing osteitis appears as a radiopacity at the periapical region of the tooth, and it is a periapical inflammatory reaction to an odontogenic infection [32]. The associated tooth may be carious or have a restoration, and usually presents with a non-vital pulp. Exostoses (bone prominence) or tori can appear radiopaque on radiographs. However, they can easily be identified on clinical examination as the cause of the radiopacity. Hypercementosis is a condition characterized by the excessive buildup of cementum on the roots of a tooth/teeth [33]. It may be associated with symptoms such as pain. Etiology may be idiopathic, secondary to local factors such as occlusal trauma, unopposed/non-functional tooth, or as a manifestation of systemic conditions such as Paget disease of the bone, acromegaly, calcinosis, or pituitary gigantism [33]. Radiographically, the radiopacity appears bulbous, following the outline of the roots of the tooth/teeth, preserving the periodontal ligament space [33]. Cementoblastoma is a neoplastic lesion of the cementum. It may be associated with jaw pain and swelling and usually arises in association with the mandibular first molar [34]. Radiographically, this neoplasm appears as a radiopaque mass attached to one or more roots of a tooth and is surrounded by a radiolucent rim [34]. Since cementoblastoma will continue to increase in size, surgical excision of the mass is encouraged, even if asymptomatic. MRONJ may present as homogeneous radiolucent areas of the jaw. However, a clinical history of antiresportive or antiangiogenic medication use will help in making the diagnosis.

The utility of cone beam computed tomography (CBCT) in the diagnosis and evaluation of COD lesions has been examined by several studies [35,36,37,38,39]. These studies have largely shown CBCT to be valuable in assessing the COD relationship to adjacent structures but have not demonstrated significant diagnostic superiority over conventional two-dimensional imaging, which has the advantages of lower radiation exposure for the patient, wider availability, and lower costs [35,36,37]. The emergence of low-dose CBCT protocols for dental applications may prove to be useful in the diagnosis and follow-up of suspected COD lesions, but such protocols have not been studied for assessing intrabony lesions [40]. If early COD is a consideration for a periapical radiolucency in association with a previously endondontically treated tooth, CBCT may be a valuable tool in assessment and diagnosis, particularly if the tooth did not have a prior history of local pain [38]. For asymptomatic cases of COD, conventional panoramic or periapical radiographs are sufficient for the diagnosis and follow-up of most cases [29,35].

Surgical intervention is not recommended for any form of asymptomatic COD. Management of asymptomatic COD consists of routine radiographic exam, along with support of proper oral hygiene practices and regular professional dental care.

## 5. Conclusions

We analyzed the demographic and clinical features of 191 new cases of COD. This reaffirms that this condition primarily affects middle-aged females of African descent and occurs most commonly in the mandible [2,3,5,6,7,39]. FLCOD is the form of COD that presents the most frequently with symptoms, and diagnosing FCOD and PCOD remains a challenge for dentists.

## Figures and Tables

**Table 1:** Summary of the demographic features of patients diagnosed with cemento-osseous dysplasia.

	All COD	Florid COD	Periapical COD	Focal COD
Gender				
Male	178 (93.2%)	82 (96.5%)	55 (87.3%)	41 (95.3%)
Female	13 (6.8%)	3 (3.5%)	8 (12.7%)	2 (4.7%)
Age distribution				
10–19	2	-	-	2
20–29	9	2	3	4
30–39	36	5	22	9
40–49	31	18	6	7
50–59	45	17	17	11
60–69	39	23	11	5
70–79	22	16	2	4
80–89	6	3	2	1
90–99	1	1	-	-
Ethnicity				
African American	160 (83.8%)	85 (100%)	53 (84.1%)	22 (51.2%)
Caucasian	24 (12.6%)	-	7 (11.1%)	17 (39.5%)
Hispanic	4 (2.1%)	-	2 (3.2%)	3 (7%)
Asian	3 (1.6%)	-	1 (1.6%)	1 (2.3%)

**Table 2:** Summary of the symptoms of patients diagnosed with cemento-osseous dysplasia.

Case No.	Age	Gender	Ethnicity	COD Type	Symptom (s)
1	66	F	AA	Florid	Draining fistula/discharge
2	79	F	AA	Florid	Exposed bone
3	60	F	AA	Florid	Exposed bone
4	63	F	AA	Florid	Pain, abscess, and failed implant
5	41	F	AA	Florid	Draining fistula/discharge and pain
6	52	F	AA	Florid	Non-healing extraction sites
7	66	F	AA	Florid	Pain
8	84	F	AA	Florid	Pain
9	48	F	AA	Florid	Pain
10	44	F	AA	Florid	Pain
11	65	F	AA	Florid	Pain and swelling
12	67	F	AA	Florid	Pain and bone exposure
13	42	F	AA	Florid	Pain and expansion
14	63	F	AA	Florid	Pain and foul discharge
15	57	F	AA	Florid	Pain and swelling
16	64	F	AA	Florid	Pain
17	54	F	AA	Florid	Pain
18	68	F	AA	Florid	Pain
19	59	F	AA	Florid	Pain and swelling after extractions
20	72	F	C	Focal	Pain and purulent discharge
21	73	F	C	Focal	Pain and swelling
22	72	F	C	Focal	Pain and swelling
23	63	F	AA	Focal	Pain and swelling
24	59	F	C	Focal	Intermittent pain
25	60	F	AA	Focal	Pain and swelling
26	62	F	AA	Focal	Pain
27	54	F	C	Focal	Intermittent pain and pressure
28	59	F	AA	Periapical	Redness and swelling

F—female, AA—African American, C—Caucasian, COD—cemento-osseous dysplasia.

## Data Availability

Data are unavailable due to privacy or ethical restrictions.

## Abbreviations

COD	cemento-osseous dysplasia
FLCOD	florid cemento-osseous dysplasia
PCOD	periapical cemento-osseous dysplasia
FCOD	focal cemento-osseous dysplasia
MRONJ	medication-related osteonecrosis of the jaw
UTHSC	University of Tennessee Health Science Center
AA	African American
C	Caucasian
H	Hispanic
A	Asian
F	female
BFOL	benign fibro-osseous lesion
OF	ossify fibroma
CBCT	cone beam computed tomography
